# New multi-injection commutation topology for circuit breakers of HVDC transmission lines

**DOI:** 10.1038/s41598-024-53832-4

**Published:** 2024-02-12

**Authors:** Fady Wadie, Mahmoud Elsisi, Tamer Eliyan

**Affiliations:** 1 Mechatronics and Robotics Engineering Department, Faculty of Engineering, Egyptian Russian University, Badr City, 11829 Egypt; 2 Department of Electrical Engineering, National Kaohsiung University of Science and Technology, Kaohsiung City, 807618 Taiwan; 3 Department of Electrical Engineering, Faculty of Engineering at Shoubra, Benha University, Cairo, 11629 Egypt; 4 Department of Electrical Power and Machines Engineering, The Higher Institute of Engineering at El-Shorouk City, Alshorouk Academy, Cairo, 11837 Egypt

**Keywords:** Transmission lines, Protection systems, Circuit breakers, Commutation system, Energy science and technology, Engineering

## Abstract

Power-sharing between countries has an essential effect on increasing the power system’s reliability and allowing a resilient energy market. High voltage direct current (HVDC) transmission systems are preferable for long-distance transmission to decrease power losses. However, HVDC transmission lines have many protection challenges including the differentiability between various types of HVDC circuit breakers (HVDC-CB). Although mechanical HVDC-CBs suffered from long response time, they superseded their solid-state counterparts in terms of price and power losses. In this paper, a multi-injection commutation system MICS-HVDC-CB is developed to provide economic and fast response HVDC-CB. The proposed breaker doesn’t add external elements to avoid any price increase but instead modifies the existing topology. The MICS consists of multiple L–C commutation circuits inserted sequentially following the receiving of the tripping signal. The proposed MICS-HVDC-CB was tested upon a real transmission line using ATP simulation software. The results emphasize that the developed MICS-HVDC-CB decreased the arcing time to 38.5% and 20% compared to passive and active DC-CBs. The impact of cooling power, arcing time constant, and fault resistance was also investigated. The results showed the effectiveness of the proposed MICS topology in reducing the arcing time while keeping a simple and economic breaker structure.

## Introduction

Recently, energy sharing between countries has been increased to support the reliability of the electrical grids and feeding remote areas. The power-sharing between countries effectively impacts the resiliency of the electrical grids and the investment in renewable energy. Thus, the interconnection has many challenges due to power losses that increase with long-distance transmission^1,2^. High voltage direct current (HVDC) transmission has been extending globally in recent decades^3–5^. That increased the interest in HVDC transmission for their pivotal role in connecting unsynchronized power systems and their ability to transmit bulk amounts of power through long distances^6,7^. The thrive in HVDC systems has increased the focus on HVDC protection systems, specifically on HVDC circuit breakers (CB)^8,9^. However, HVDC faces a more significant challenge than their AC counterparts due to the absence of naturally commuting current zero crossing that allows circuit breakers to interrupt the arc. For this reason, HVDC circuit breakers (CBs) utilize additional circuits to force current with zero crossing^10,12^. Generally, HVDC CB consists of an interrupting element, either gaseous or vacuum, and a parallel commutating L–C filter circuit responsible for forcing the current with zero crossing^11,13,16^. The L–C branch could be used such that the capacitor is not charged prior to operation and in that case the CB is considered to be passive CB. If the capacitor was charged prior to operation, that CB is termed as active CB. Active CB is considered more efficient in suppressing the arc in lesser time than passive CB. That is because of the injected capacitive current that imposes the arc in active CB. Additional shunt branches include an R–C circuit and metal–oxide varistor (MOV). The R–C addition is utilized to limit the rate of rise of recovery voltage (RRRV). At the same time, the MOV is used to restrict the transient recovery voltage (TRV) and to absorb the excess energy following the interruption of the arc^15–18^.

The interrupting element, vacuum or SF6, could be modeled according to models presented in the literature^19,22^. For the SF6 interrupter, the modeling of their arc extinguishing sequence could be done according to the physical or black box arc models. While the physical model provides more detailed and complex modeling for the arc interruption process, the black box model focuses on the change of conductance of the arc^23,26^. The most widely adopted black box models include the Cassie and Mayr dynamic arc equations^11,13,16^. In this study, the Mayr model will be utilized to describe the mechanism of the SF6 interrupter. The enhancement of the HVDC CB performance was highly investigated in literature as summarized in Table 1.

**Table 1:** Summary of literature.

Enhancement proposed	^11^	^27^	^29^	^30^	^31^	^33^	^34^	^35,36^	^37^	^38–40^	^14,41–43^
Employment of superconductor to limit fault current	✓	–	–	–	–	–	–	–	–	–	–
Analysis of the impact of fault current limiters upon DC CB	✓	✓	✓	–	–	–	–	–	–	–	–
Application of resistive superconducting fault current limiters	–	–	✓	–	–	–	–	–	–	–	–
Extended H-bridge topology for DC CB	–	–	–	✓	–	–	–	–	–	–	–
Capacitor commutated DC CB topology	–	–	–	–	✓	–	–	–	–	–	–
Employment of Supercapacitors	–	–	–	–	–	✓	–	–	–	–	–
Damping current commutation	–	–	–	–	–	–	✓	–	–	–	–
Coupled inductor based HVDC Breaker	–	–	–	–	–	–	–	✓	–	–	–
Inverse current injection technique	–	–	–	–	–	–	–	–	✓	–	–
Passive resonance HVDC Breakers	✓	✓	✓	–	–	–	–	–	–	✓	–
Active resonance HVDC Breakers	–	✓	✓	–	–	–	–	–	–	–	✓

The previous table shows that literature had succeeded in enhancing the performance of HVDC CB by reducing the arcing time and the fault current. In^11^, a superconducting element was combined with mechanical DC-CB to analyze the performance and transient behavior of the beaker during faults using EMTP software. Analysis for the sizing process for fault current limiters combined with DC-CB was done in^27^ using PSCAD and MATLAB soft-wares. Superconducting fault limiters was combined with various types of HVDC CBs to analyze their impact on the resulting transient behavior in in^29^ The benefits from the extended H-bridge design were utilized in^30^ where bidirectional current flow through main circuit breaker could be achieved allowing a reliable performance of breaker as confirmed by simulation results using PSCAD^30^. A modified hybrid HVDC combining a capacitor commutated topology proved to be cost effective and reliable in^31^. Compensation for reactive power using supercapacitors during switching events was investigated in^33^. Integrating both current commutation and damping procedures were simulated in^34^. Another perspective was proposed in^35^ by dividing the breaker into main breaker and branch circuit breaker with flexible number of inductors enhancing the limiting capability of arc current. A similar approach was proposed in^36^ where coupled inductor and capacitor were used to form a resonant circuit during the fault. To provide an artificial zero crossing of fault current. For current interruption of bidirectional faults, an inverse current injection technique was proposed in^37^. This technique utilizes both diode and capacitor to prevent the current from changing their polarities during faults allowing the minimization of interruption time. Passive and active resonance DCCB were commonly employed types of circuit breakers in literature^38–43^. In^38^, a passive resonance circuit breaker modeled with black box arc model was studied to determine the interruption capability curve. Alternatively, active resonance circuit breakers differ from their passive counter parts in requiring pre-charging of capacitors used. This imposes a challenge as these pre-charging circuits are complicated^41^. To overcome this problem, a self-charging scheme that utilizes the induced voltage from the current limiting reactor to charge the commutation capacitor was proposed in^41^.

Finally, it could be summarized that the literature is rich with proposed topologies that intend to enhance the current limiting capability and still be cost effective. However, most of them had to use additional elements to reach that result making the HVDC CB more expensive and complicated in structure. Therefore, the main problem that this paper tends to address is to propose a new topology for HVDC CB that does not add additional elements to its topology and yet still reaches a satisfactory performance in terms of arcing time, TRV, and RRRV. The proposed topology will rely on modifying the components of the L–C commutation to increase their ability to reach a current with zero crossing faster. In addition, the modified L–C will not require the presence of an R–C circuit, allowing a much more straightforward and cheaper breaker topology. The proposed modifications include the usage of power ceramic capacitors in the L–C filter capable of being charged to high voltage values up to 100 kV^44^. These power capacitors will be pre-charged before operation and connected in reverse with the expected current flow within the L–C circuit, allowing them to inject a high capacitive current in reverse to fault current during faults. That type of topology is typically known as active HVDC CB^5^. The novelty of the proposed scheme relies on using multiple L–C filters that are inserted sequentially after the instant of tripping the CB. In addition, the reliance upon ceramic capacitors within L–C filters allows the injected capacitive currents to be of high value bringing the zero-crossing current much faster. Therefore, the contributions of this paper will be:

- Proposing a new topology for HVDC CB that utilizes the multiple ceramic capacitor-based L–C filters that are inserted sequentially after tripping the CB.
- The proposed topology injects high capacitive currents allowing the reach for zero crossing much faster, reducing the arcing time, the TRV, and RRRV.
- The main novelty of the proposed topology is accomplishing the previous results mentioned in point 2 while using simple and economic topology. Hence, the proposed topology allows mechanical CB to overcome its main drawback of long arcing time while still having the advantage of being economical.
- Assessing the impact of the different parameters upon the performance of the proposed topology for HVDC CB by undergoing a parametric investigation.

Therefore, the main focus of this paper is switching transients within the DC side of the HVDC system and for such reason the injected harmonics of power electronic based converters within the AC side will not be discussed. Such a trend in focusing upon DC side was previously adopted in literature in^7–11,11–14,14–43^. The rest of this paper is organized as follows. “The proposed HVDC CB topology” Section presents the proposed topology for the HVDC CB. “Modeling of the system” Section presents the modeling of the SF6 current interrupter using the Mayr model upon ATP/EMTP software. The usage of simulation platforms is considered widely common practice in literature as previously mentioned in review in^11,14,27,29–43^. These simulation platforms have proven to be give a reliable and trustable results as accepted in research community. In “Simulation results” Section, the proposed HVDC CB is used to protect a real transmission line in Egypt. An investigative parametric analysis is undergone in the same section to analyze the impact of the variation of the breaker parameters models upon the performance of CB. Finally, comparative assessment and conclusions are drawn in “Comparative assessment of MICS-HVDC-CB” and “Conclusions” Sections, respectively.

## The proposed HVDC CB topology

**Figure 1:**
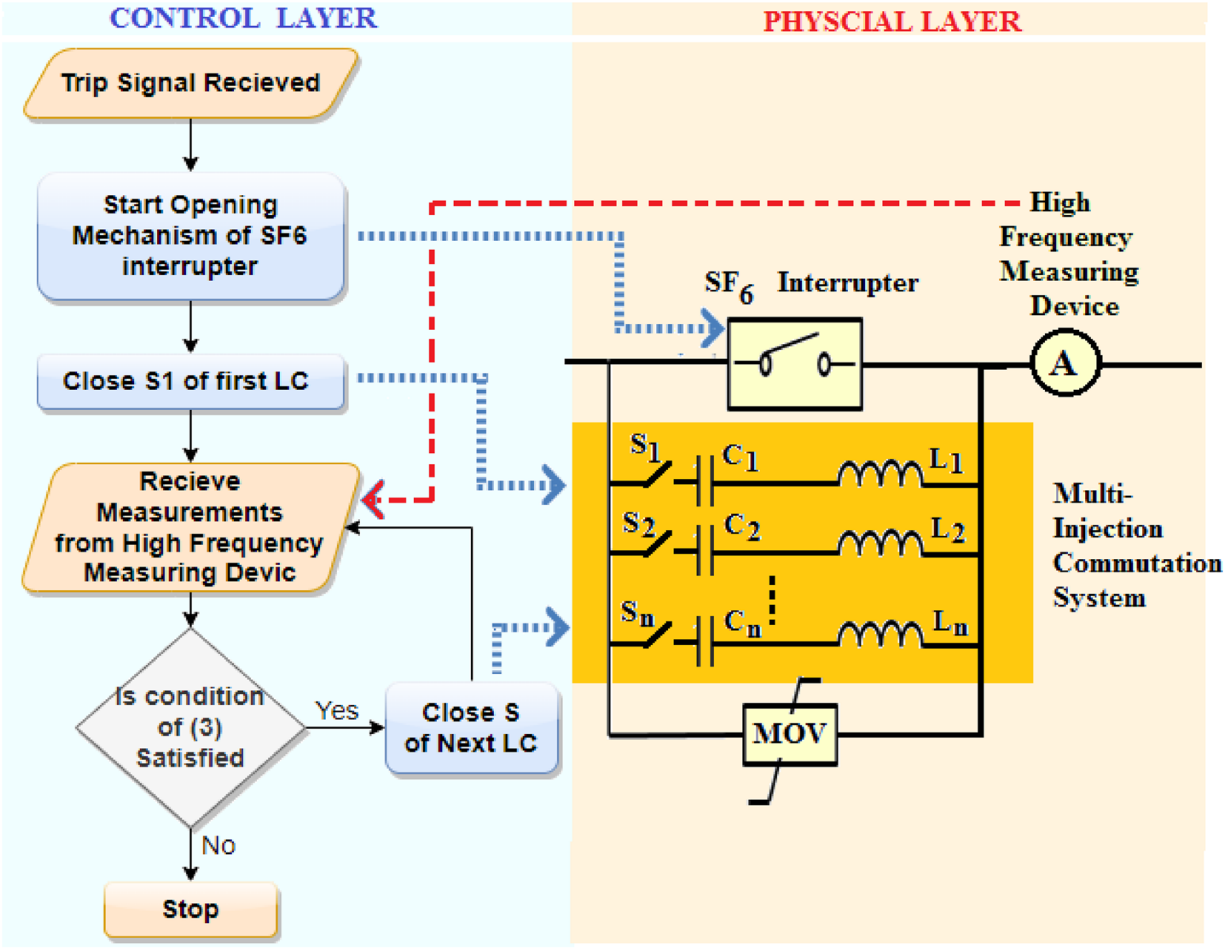
Layout of MICS-HVDC-CB.

The conventional topology of HVDC CB consists of a mechanical interrupter, shunt L–C branch, shunt R–C branch, and MOV. The L–C branch is controlled using a switch that remains open during healthy operation and closes at the instant receiving the tripping signal to force an oscillating current into the circuit. The imposed oscillating current brings the breaker’s current to a forced zero crossing allowing the interrupter to extinguish the arc at that instant. A TRV arises at that moment that is limited by the MOV till the current reaches zero. The proposed topology differs in using multiple L–C branches that are inserted sequentially till the arc is suppressed while using a control algorithm to control the number of inserted L–C branches as shown in Fig. 1.

**Figure 2:**
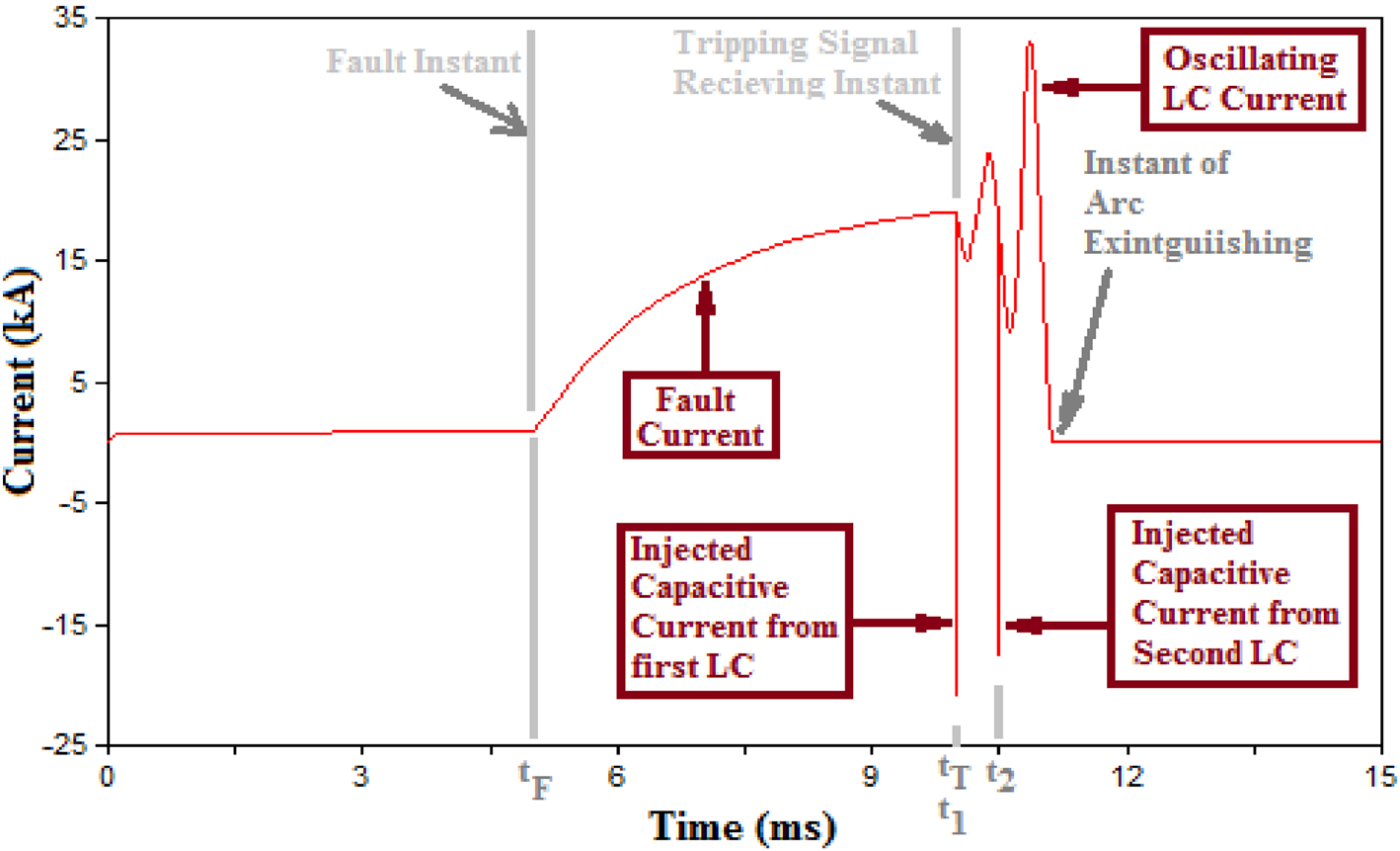
Current through SF6 mechanical interrupter.

The main features for MICS-HVDC-CB layout are summarized into four main points as follows:

- Multiple L–C circuits are used instead of a single L–C circuit. To furtherly explain the reason behind using multiple L–C branches, the effect of each LC branch is first stated. For each Pre-charged L–C branch inserted during the fault, a high pulse of capacitive current is injected in a path opposite to the arc current leading to significant reduction in arc current. Following that injected current, an oscillating current is generated from the L–C circuit. The oscillating current lasts till a forced current zero crossing is reached in the interrupter and the arc is suppressed. Both the injected capacitive current that appears like a pulse and the following oscillating current are shown in Fig. 2. It could be concluded that if additional capacitive current is injected, that would have a higher reduction effect upon the arc current allowing the following oscillating current to reach a zero crossing much quicker. For such reason, multiple L–C branches are used to be inserted on a step-by-step basis to inject capacitive current pulses till the arc is suppressed as shown in Fig. 2 which uses two L–C branches injecting two successive capacitive currents. The LC branches are inserted in steps and not all at once to avoid the injection of a massively high capacitive current that was not needed for the arc current in this case. As when L–Cs are inserted in steps, once the injected current is high enough to suppress the arc, no additional L–Cs are inserted. The number of the required L–C circuits would depend on the capacitors' rating and the system's expected fault currents. The designer of the breaker would select the number of needed L–C to ensure that their injected currents would be enough to extinguish the maximum expected fault current.
- The capacitor of L–C branch is selected to be a power ceramic capacitor so that it can bare the charge from high-voltage systems.
- The timing of insertion of the L–C circuit is selected based on the following criterion. The first L–C is inserted at the tripping instant. The second L–C will only be inserted if the fault current remains not extinguished. The time of insertion of the second L–C will be equal to the period of the oscillating cycle of the L–C filter that is given in (1). That criterion is chosen so the injected currents would have the same effect regarding their direction concerning the oscillating current as they are separated by one oscillating cycle. If the fault current remains unextinguished, a third L–C is inserted after one oscillating cycle from the second L–C. The sequence is repeated until the arc is extinguished or the full stack of L–C circuits is inserted. The general formula for the timing of insertion of each L–C is given in (2).
  1 $${t}_{2}={t}_{1}+T= {t}_{1}+(2\pi \sqrt{LC} )$$
  2 $${t}_{n}={t}_{n-1}+T= {t}_{n-1}+(2\pi \sqrt{LC} )$$
  where ${t}_{1}$ is the instant of connection of the first L–C circuit that is equal to the time of receiving the tripping signal. ${t}_{2}$ is the instant of connection of the second L–C. ${t}_{n}$ and ${t}_{n-1}$ are the timing of insertions of L–Cs number “n” and “n-1”, respectively. $T$ is the periodic time of the oscillating L–C circuit that is equal to $2\uppi \sqrt{{\text{LC}}}$.
- A control algorithm is used to control the timing of the insertion of L–C. The algorithm receives from a signal high-frequency measuring device that measures the current flowing through the breaker as shown in Fig. 1. The measured current value is compared to the last reported measured current value inside the algorithm so that if the current is increasing, that indicates the faults are still sustained. If the current values are decreasing, the arc has been extinguished, and the MOV would suppress the remaining current; hence, no additional insertions of L–C circuits are required. The criterion for insertion of new L–C is given in (3).
  3 $$\mathrm{If }({I}_{t}- {I}_{t-1}\ge zero) \&\mathrm{ \,the \,timing \,condition \,in \,}(2)\mathrm{ \,is \,reached \,then \,connect \,next \,L}-{\text{C}}$$
  where, ${I}_{t} and {I}_{t-1}$ are two consecutive current measurements reported from the high frequency measuring device measuring the breaker current.

The previous modifications rely on modifying the components of the breaker without adding extra elements to avoid the increase in breaker’s price. In addition, the proposed changes allow the breaker to reduce the arcing time, significantly overcoming the main challenge for mechanical DC CB.

It could be summarized that the proposed topology layout consists of two layers, the control layer, and the physical layer. The physical layer contains multiple L–C commutation circuits, which employ power ceramic capacitors, a solid-state switch to control the entrance time of the L–C circuit, and a high-frequency measuring device to monitor the current through the CB. The control layer contains the algorithm that controls the timing of connecting the L–C. The layers, the control algorithm's flow chart, and the data exchange between layers are shown in Fig. 1. The proposed layout could be named a multi-injection power ceramic capacitor-based commutation system. The multi-injection part of the name is based on using multiple capacitors that inject capacitive currents. The term commutation system is used as the L–C circuits are controlled via a control algorithm that defines their need for insertion or not and the timing for their insertion. Finally, the acronym for the proposed topology should be MI-PCCB-CS which are the initials for all the words of the name. For the sake of simplicity, the topology could be named a multi-injection commutation system HVDC CB or MICS-HVDC-CB.

At this point, the frame of operation of the topology could be summarized in chronological order as follows and as shown in Fig. 2:

Step 1: ${\mathbf{t}}_{\mathbf{F}}$ = **Fault instant:** the fault is sensed, and a tripping signal is sent to the breaker.
Step 2: ${\mathbf{t}}_{\mathbf{T}}$ = **Tripping instant:** the tripping signal allows the tripping mechanism of the mechanical interrupter to operate. In addition, the signal is also sent to the switch connecting the first L–C circuit to allow its insertion into the circuits.
Step 3: ${\mathbf{t}}_{\mathbf{T}}$ < **t < **(${\mathbf{t}}_{\mathbf{T}}$** + **$2{\varvec{\uppi}}\sqrt{\mathbf{L}\mathbf{C}})$** :** The high frequency measuring device sends its current measurements continually to the control algorithm within the breaker which decides whether a new L–C will be inserted or not according to the criterion mentioned previously in (3).
Step 4: **t = **(${\mathbf{t}}_{\mathbf{T}}$ **+** $2{\varvec{\uppi}}\sqrt{\mathbf{L}\mathbf{C}})$: Assuming that the criterion of (3) is satisfied, a new L–C is connected.
Step 5: Steps 3 and 4 will be repeated until the criterion in (3) is no longer satisfied and the arc will be extinguished at that instant.

## Modeling of the system

### Modeling of the SF6 interrupter

The main block within the model of the breaker is Mayr’s black-box model of the SF6 interrupter. Mayr’s model defines the CB’s ability to reach a successful opening based on dynamic analysis for the arc^11^. These analyses depend on computing the variable conductance of the arc, which consists of four sub-stages, each representing a specific transition process of the breaker from the closed state to the open state. These stages are defined as follows: closed breaker, arcing, arc extinguishing, and open stages^45,46^. The model is considered as a constant resistor with negligible value of 1 μΩ and high value of MΩ for closed and opened sub-stages, respectively. While for the arcing and the extinguishing sub-stages, a series connection of the Mayer arc model could be deduced as follows^13,45^.

- From the energy point of view, an imbalance exists between heating power involved with the arc ${(P}_{H})$ and cooling power as a result from the energy dissipated from the arc ${(P}_{o})$*.* That leads to an amount of energy to be stored within the arc column *Q(t)* as given in (4).
  4 $$\frac{dQ(t)}{dt}={P}_{H}- {P}_{o}$$
- The arc conductance ${g}_{m}\left(t\right)$ could be represented in terms of the stored energy *Q(t)* as given in (5) where τ is the arc time constant.
  5 $${g}_{m}\left(t\right)=K \frac{Q(t)}{{P}_{o} \tau }$$
- The formula in (4) could be rewritten in terms of the arc conductance as in (6).
  6 $$\frac{dQ(t)}{d{g}_{m}}\frac{d{g}_{m}}{dt}={P}_{H}- {P}_{o}$$
- The mathematical expression in (5) could be substituted in (6) while equating the heating power to the amount of electrical power from the arc (*v *$\times$* i*), where *v* is the arc voltage, and *i* is the arc current, we get the equation in (7).
  7 $$\frac{{P}_{o} \tau }{{g}_{m}} \frac{d{g}_{m}}{dt}=(v\times i)- {P}_{o}$$
- Finally, Eq. (4) could be rearranged while considering the conductance ${g}_{m}=v/i$ in (85).
  8 $$\frac{d{g}_{m}}{dt}=\frac{1}{\tau }\left(\frac{{i}^{2}}{ {P}_{o}}- {g}_{m}\right)$$

**Figure 3:**
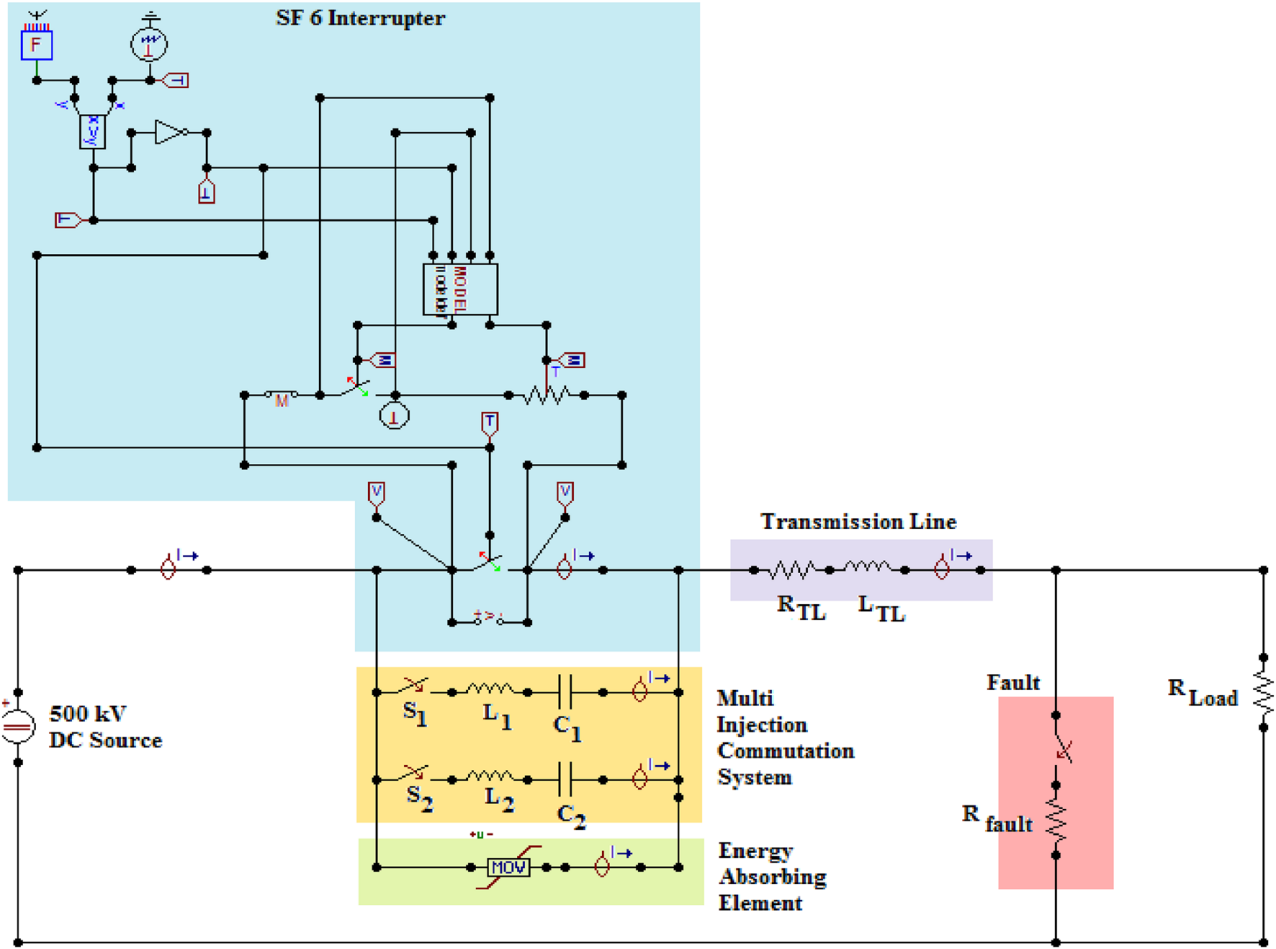
Testing System for MICS HVDC CB using ATP/EMTP.

The main variables within Mayr’s arc model are the arc time constant (τ) and the coefficient of cooling power (${P}_{o}$). The MODELS component was used to model the SF6 interrupter upon ATP/EMTP software. This component helps build a coded program to gather its inputs from the electrical signals within the simulated system. Its output signals could be used to control components within the system. The mathematical formula in (7) is built using coded software within the MODELS component. The SF6 interrupter model and the testing system are shown in Fig. 3.

### Modeling of the multi-injection commutating system

The commutation branches are built using L and C components with values set to 0.3 mH and 20 µF, respectively. Each branch of the system is used to control its time of insertion according to the criterion in (3). The capacitances are pre-charged with a percentage of the system voltage based on the following points:

1. The value of the expected fault current in the system.
2. The selected charging value should inject a suitable capacitive current relative to the fault current in the first point.
3. The voltage rating of the capacitors.
4. The first two point were analyzed by the authors for the selected testing system, and it was found that charging the capacitors to 1% of the system voltage was capable of injecting suitable currents during faults. To satisfy the last point, power ceramic capacitors were used, capable of baring up to 100 kV^44^.

### Modeling of the energy absorbing element

The final component of the HVDC CB is the energy-storing element which is usually a metal-oxide varistor (MOV). The MOV is connected in parallel with the interrupter and the commutation system. The employment of MOV is beneficial for its non-linear I-V characteristic allowing limited post arc TRV and store the excess energy generated from the fault incident^13^.

### Modeling of the testing system

**Figure 4:**
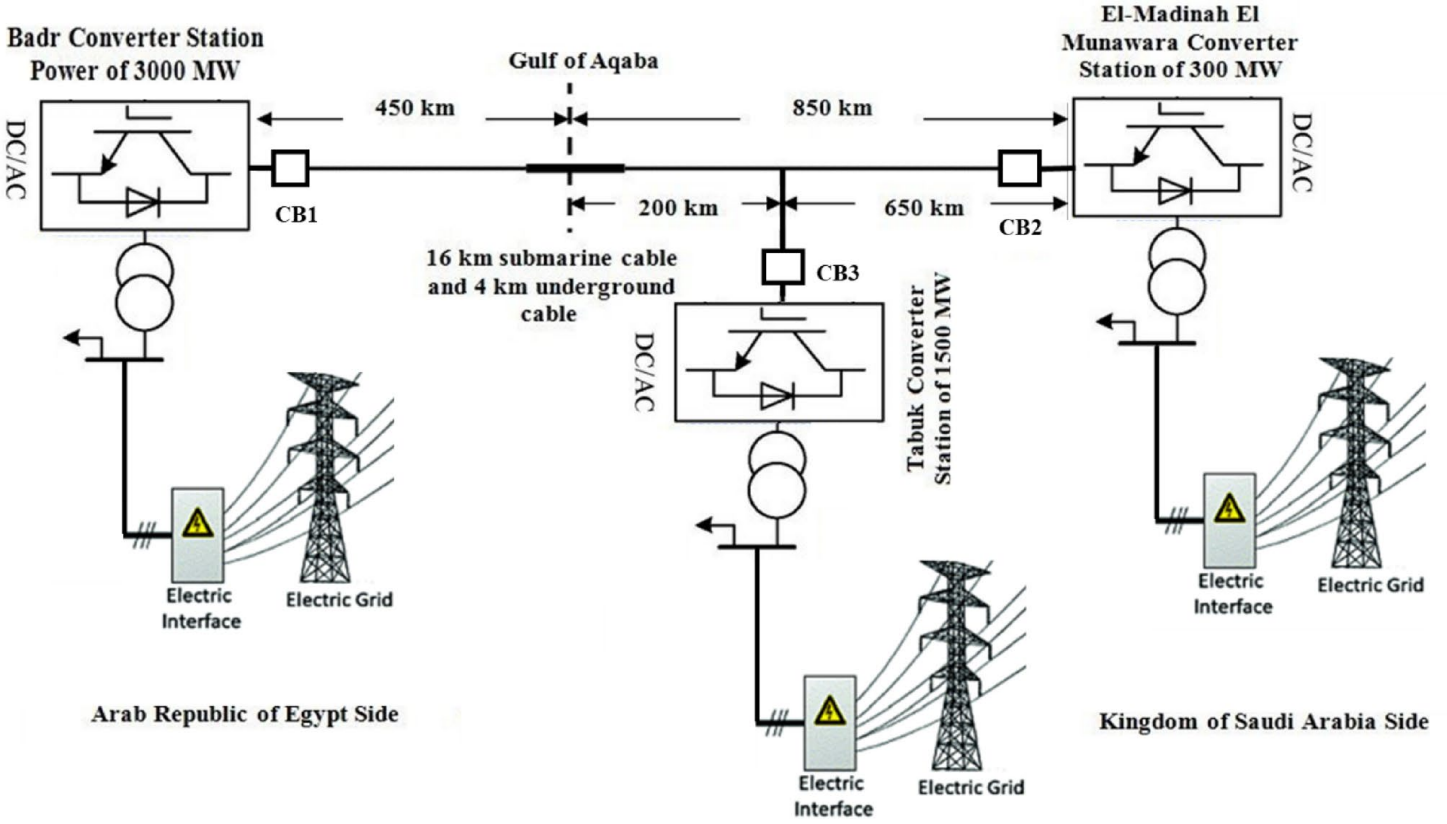
The HVDC transmission representation of the studied practical system between Egypt and Saudi Arabia.

The testing system selected is a real 450 km overhead DC transmission line located in Egypt and connects Badr substation and Elnabaq switching station presented in^16^. The system was modeled using ATP/EMTP software. The selected system is considered a portion of an HVDC interconnection system between Egypt and the Kingdom of Saudi Arabia, which consists of two 500 kV AC/DC substations in Badr City, a linking station, and a 1,300 km transmission line to Medina and Tabuk in Saudia Arabia as shown in Fig. 4. The selected testing transmission line is 450 km overhead DC transmission line located in Egypt and connects Badr substation to Elnabaq switching station. A 500 kV HVDC source is used to model the selected system to provide a power supply to 650 Ω load. The 450 km DC overhead transmission line connecting the source and the load is modeled as a 24.5 Ω resistor and 45 mH inductor, as shown in Fig. 3.

## Simulation results

The MICS HVDC CB was used to protect the HVDC transmission line within the system presented in “Modeling of the testing system” Section. To examine the efficacy of the proposed MICS, two testing sequences will be applied. The first sequence will include creating a fault across the line and comparing the results for MICS, conventional active HVDC CB, and passive HVDC CB. The second sequence will focus on the impact of various parameters on the results of MICS-HVDC CB.

### Testing sequence 1: comparison between passive, active, and MICS HVDC CB

**Figure 5:**
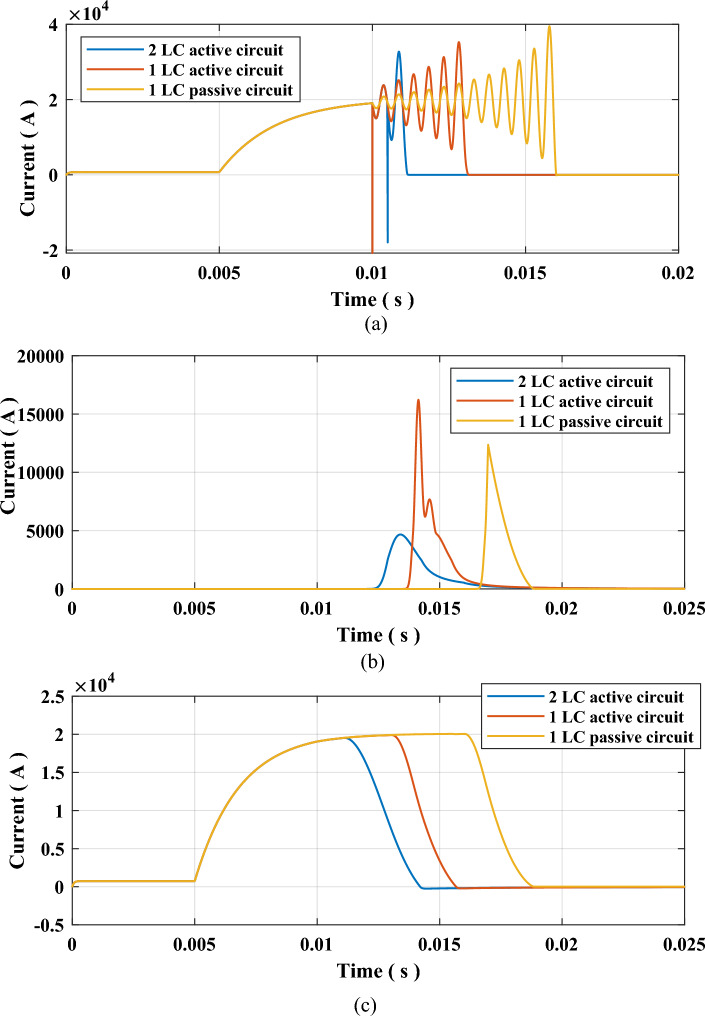
Waveforms of currents due to testing sequence 1 (**a**) Current through SF6 Interrupter, (**b**) Current through MOV, and (**c**) Current through the system.

**Figure 6:**
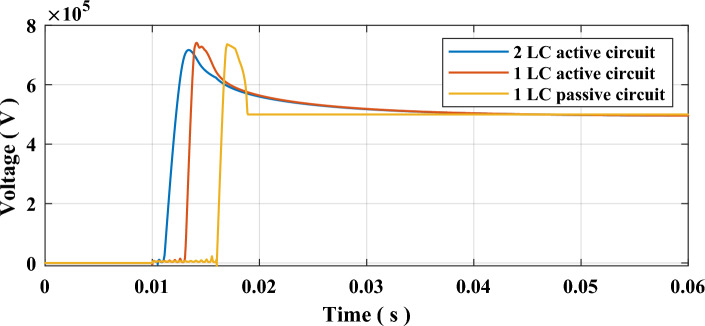
Voltage across SF6 interrupter contacts due to testing sequence 1.

A fault will be inserted for this testing sequence, as shown in Fig. 3 at 5 ms. The fault resistance is 0.1 Ω to allow a high fault current across the system. The cooling power ${P}_{o}$ of the SF6 interrupter set 100 MW and arc time constant τ set to 10 µs. As mentioned earlier, the L and C of commutation branches are set to 0.3 mH and 20 µF. This first simulation case, within this sequence, was done for a passive HVDC circuit breaker with only one commutation circuit and with non-charged capacitor. The second case was done for an active HVDC circuit breaker with only one commutation circuit, and its capacitor is pre-charged to 5 kV. The third and final case is for MICS-HVDC-CB, which contains two commutation circuits that are pre-charged to 5 kV. The first L–C is inserted at 10 ms when receiving the tripping signal. The second L–C is inserted at 10.5 ms, according to (2). The results of all three cases are presented in Table 2 and shown in Figs. 5 and 6. The results show that MICS could reduce the arcing to 1.239 ms from 3.127 ms for active and 6.117 for passive HVDC CBs. It could be noticed that MICS has reduced the arcing time to 38.5% and 20% when compared to passive and active HVDC CBs. The TRV was decreased slightly, while RRRV was almost halved. Such results indicate the remarkable performance of MICS-HVDC-CB when compared to other mechanical-based HVDC-CB.

**Table 2:** Results of testing sequence 1.

Type of HVDC CB	Arcing time (ms)	TRV (kV)	RRRV (kV/µs)	Clearing time (ms)
Passive (1 L–C)	6.117	736.07	9.7556	8.91
Active (1 L–C)	3.217	741.00	9.4360	5.85
MICS (2 L–C)	1.239	716.85	4.2439	4.34

### Testing sequence 2: impact of different parameters upon MICS performance

To examine the effect of various parameters upon the performance of MICS-HVDC-CB, the parameters will be varied independently, and their results will be evaluated. These parameters include the cooling power of the SF6 interrupter, the arc time constant, and the fault resistance. The fault will remain to be inserted at 5 ms. Each of the previous parameters will be tested in separate cases with other parameters fixed as follows.

### Impact of cooling power of SF6 interrupter

**Figure 7:**
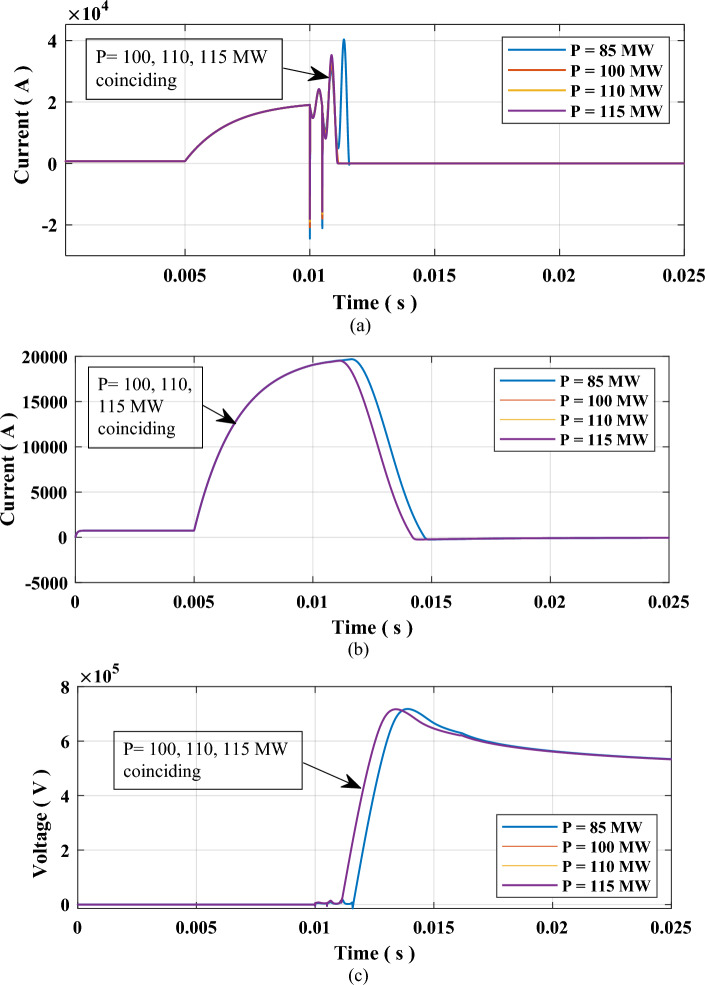
Results due to the impact of cooling power (**a**) Current through SF6 Interrupter, (**b**) Current through the system, and (**c**) Voltage across SF6 interrupter contacts.

The cooling power ${P}_{o}$ of the SF6 interrupter will be varied from 85 MW up to 115 MW. The arc time constant τ and fault resistance will remain fixed at 10 µs and 0.1 Ω, respectively. The results for this case are presented in Table 3 and Fig. 7. The results show that for cooling powers greater than 100 MW, there was no impact on the performance of the CB. That is considered a great advantage for the MICS-HVDC-CB, as it would be enough to reach great performance with only 100 MW cooling power allowing a reduction in cost for the SF6 interrupter.

**Table 3:** Results of testing sequence 2: impact of the cooling power of SF6 interrupter.

${{\varvec{P}}}_{{\varvec{o}}}$ (MW)	Arcing time (ms)	TRV (kV)	RRRV (kV/µs)
85	1.692	717.9	4.313
100	1.233	716.69	4.270
110	1.225	716.69	4.2729
115	1.217	716.69	4.2729

### Impact of arcing time constant

**Figure 8:**
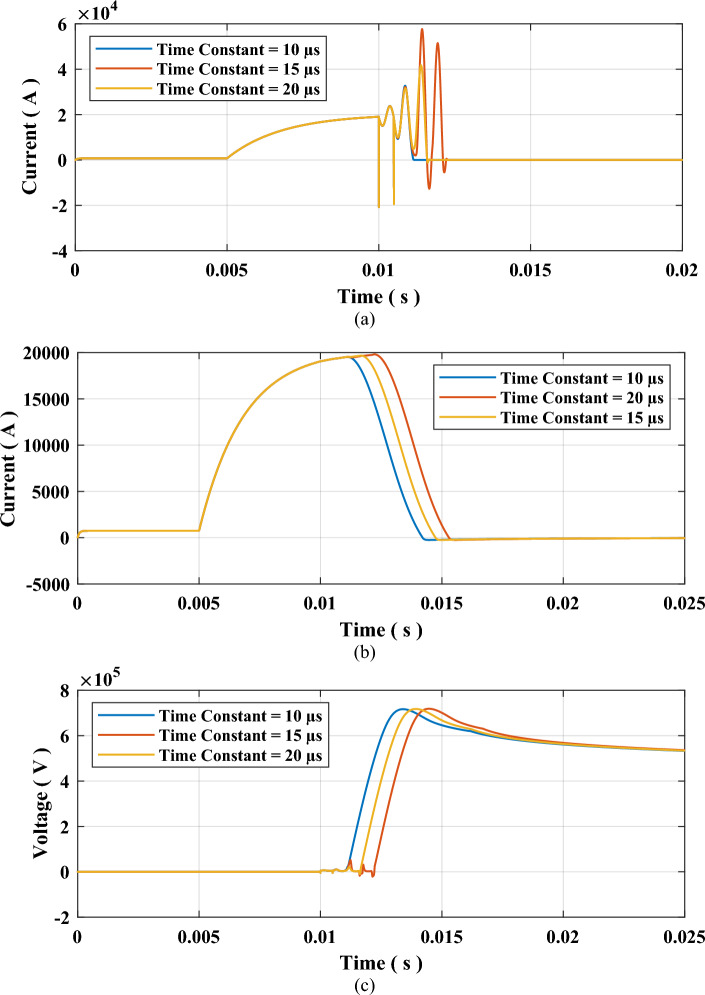
Results for testing sequence 2 impact of arcing time constant (**a**) Current through SF6 Interrupter, (**b**) Current through the system, and (**c**) Voltage across SF6 interrupter contacts.

The arcing time constant τ will be varied from 10 µs to 20 µs with other parameters fixed such that ${P}_{o}$ is set to 100 MW and the fault resistance is selected to 0.1 Ω. The results for this case are presented in Table 4 and Fig. 8. The results show that changing the time constant affected only the arcing time, while the TRV and RRRV had negligible change.

**Table 4:** Results of testing sequence 2: impact of arcing time constant.

τ (µs)	Arcing time (ms)	TRV (kV)	RRRV (kV/µs)
10	1.243	716.85	4.417
15	1.895	717.85	4.368
20	2.419	718.71	4.350

### Impact of fault resistance

**Figure 9:**
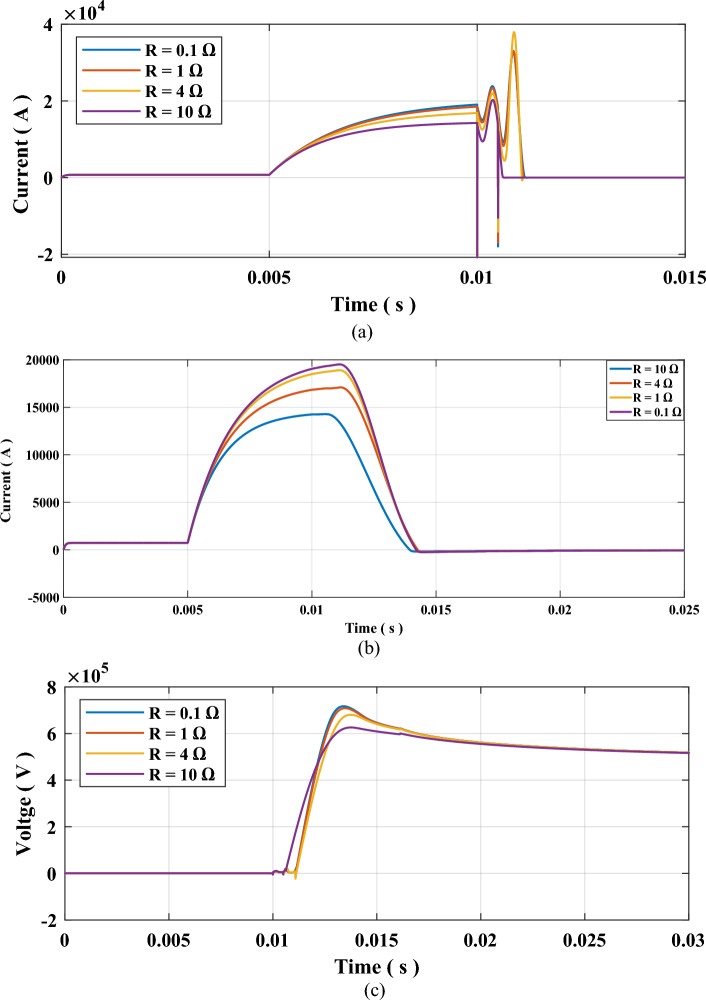
Results for testing sequence 2 impact of fault resistance (**a**) Current through SF6 Interrupter, (**b**) Current through the system, and (c) Voltage across SF6 interrupter contacts.

The effect of fault resistance is examined by varying it from 0.1 to 10 Ω while the other parameters remain constant such that ${P}_{o}$ is set to 100 MW and arcing time constant τ set to 10 µs. The results for this case are presented in Table 5 and Fig. 9. The results show that reducing the severity of the fault by increasing the fault resistance, lead to a reduction in the time required to extinguish the arc.

**Table 5:** Results of testing sequence 2: impact of fault resistance.

${\text{R}} ( \Omega )$	Arcing time (ms)	TRV (kV)	RRRV (kV/µs)
0.1	1.243	716.00	4.538
1	1.226	708.17	3.855
4	1.20	680.20	3.497
10	0.72	625.90	3.093

## Comparative assessment of MICS-HVDC-CB

The main types of HVDC-CBs include mechanical-based HVDC-CBs, solid state, and hybrid HVDC CBs^5^. The variation among those three main types is due to the difference between them in advantages and disadvantages and hence, their degree of suitability is different HVDC networks. For example, mechanical DC CBs are advantageous because of their low cost and operating resistance; on the other hand, their response is too slow. While solid-state DC CBs respond much faster, their total cost and power losses are high. Hybrid CBs intended to solve this problem but still have a non-cheap price^5^. The proposed MICS-HVDC-CB inherits the main advantages of low-cost mechanical DC CBs and negligible operational power losses. It also overcomes the main disadvantage of mechanical DC CBs, which is the slow response. The results showed that the MICS-HVDC-CB could reduce the arcing times to 1.239 ms from 3.127 ms for active and 6.117 for passive HVDC CBs which is equal to 20% in comparison to active mechanical DC CB and to 38.5% in comparison to passive DC-CB. Such enhancement in performance did not require additional elements to be included in the breaker such as current limiting elements but rather modifications of the existing components allowing the price of the MICS-HVDC-CB to be in the same range as conventional mechanical DC-CBs. A comparison between different types of HVDC CBs is presented in Table 6. From this table, it could be concluded that the main advantages of MICS-HVDC-CB are:

1. Enhancing the performance of mechanical DC-CB to be fast response HVDC-CB without increasing its price or operational increasing power losses.
2. The MICS keeps the advantage of mechanical DCCB and overcomes its main disadvantage, allowing it to be superior to solid state or hybrid CBs which would still be expensive and have high power losses compared to MICS.

**Table 6:** Comparison between different types of HVDC-CBs.

	Passive mechanical DC CB	Active mechanical DC CB	Solid state DCCB	Hybrid DCCB	MICS-HVDC-CB
Arcing time	Large	Medium	Very small	Small	Small
Price	Low	Low	High	High	Low
Power losses	Low	Low	High	High	Low

## Conclusions

DC circuit breakers have a significant role in protecting HVDC transmission systems. Various types of DC CBs have been manufactured and investigated in the literature. In this paper, a MICS-HVDC-CB was proposed. The MICS-HVDC-CB does not add any new elements to the conventional topology of mechanical DC CBs, allowing it to remain still affordable. The MICS-HVDC-CB relies on modifying the existing elements of the breaker by changing the commutation branch from a single branch to multiple branches inserted sequentially based on a control algorithm. The capacitors within the L–C branches were chosen to be power ceramic capacitors capable of being pre-charged to high voltage values. The modification of the proposed topology allows the sequential injections of high capacitive currents following the receiving of the tripping signal. That reflects a significant reduction in arcing time and a much faster response of the breaker. The MICS-HVDC-CB was tested upon a real part of 3000 MVA, 500 kV HVDC transmission between Egypt and the Kingdom of Saudi Arabia by using ATP simulation software. The results showed that MICS-HVDC-CB could reduce the arcing time to 38.5% and 20% compared to passive and active mechanical DC CBs. The impact of the parameters was also tested, including the cooling power, the arcing time constant, and the fault resistance. Finally, the performance of MICS-HVDC-CB was compared to other types of DCCBs, which is advantageous in its capability of reducing the arcing time while keeping economic topology.

## Data Availability

The data that support the findings of this study are available from the corresponding author upon reasonable request.
